# Digital clinical empathy in a live chat: multiple findings from a formative qualitative study and usability tests

**DOI:** 10.1186/s12913-024-10785-8

**Published:** 2024-03-08

**Authors:** Hanna Luetke Lanfer, Doreen Reifegerste, Winja Weber, Paula Memenga, Eva Baumann, Julia Geulen, Stefanie Klein, Anne Müller, Andrea Hahne, Susanne Weg-Remers

**Affiliations:** 1 School of Public Health, Bielefeld University, Universitaetsstrasse 25, 33615 Bielefeld, Germany; 2 German Cancer Research Center (DKFZ), Division Cancer Information Service, Heidelberg, Germany; 3 Department of Journalism and Communication Research, Hanover University of Music, Drama and Media, Hanover, Germany; 4 BRCA-Network, Cologne, Germany

**Keywords:** Clinical empathy, Empathy research, Qualitative content analysis, Usability tests, Live chat

## Abstract

**Background:**

Clinical empathy is considered a crucial element in patient-centered care. The advent of digital technology in healthcare has introduced new dynamics to empathy which needs to be explored in the context of the technology, particularly within the context of written live chats. Given the growing prevalence of written live chats, this study aimed to explore and evaluate techniques of digital clinical empathy within a familial cancer-focused live chat, focusing on how health professionals can (a) understand, (b) communicate, and (c) act upon users’ perspectives and emotional states.

**Methods:**

The study utilized a qualitative approach in two research phases. It examined the expected and implemented techniques and effectiveness of digital clinical empathy in a live chat service, involving semi-structured interviews with health professionals (*n* = 9), focus group discussions with potential users (*n* = 42), and two rounds of usability tests between health professionals (*n* = 9) and users (*n* = 18). Data were examined using qualitative content analysis.

**Results:**

Expected techniques of digital clinical empathy, as articulated by both users and health professionals, involve reciprocal engagement, timely responses, genuine authenticity, and a balance between professionalism and informality, all while going beyond immediate queries to facilitate informed decision-making. Usability tests confirm these complexities and introduce new challenges, such as balancing timely, authentic responses with effective, personalized information management and carefully framed referrals.

**Conclusions:**

The study reveals that the digital realm adds layers of complexity to the practice of clinical empathy. It underscores the importance of ongoing adaptation and suggests that future developments could benefit from a hybrid model that integrates the strengths of both AI and human health professionals to meet evolving user needs and maintain high-quality, empathetic healthcare interactions.

**Supplementary Information:**

The online version contains supplementary material available at 10.1186/s12913-024-10785-8.

## Background

Empathy is pivotal in human interactions as it fosters understanding and connection between individuals and is considered a prerequisite for prosocial behavior [1]. From an overarching perspective, scholars agree that empathy is complex and refers to several psychological states or a process rather than being a single, uniform concept [2, 3]. It is considered a multidimensional process that involves affective, cognitive, and behavioral aspects. While terminology differs, scholars have defined empathy as a threefold process: (a) *understanding* another person’s perspectives and emotional states, (b) *communicating* and (c) *acting* upon this understanding [2–4]. Traditionally, empathy has been characterized by face-to-face interactions in real-time, rich in non-verbal cues, body language, tone of voice, and immediate responses [5]. This also applies to clinical empathy, the empathy of health professionals for patients, which plays a vital role in patient-centered care. It is particularly crucial during distressing diagnoses or stressful medical experiences, as it is linked to increased self-efficacy and treatment adherence, as well as reduced emotional distress and fear among patients [6, 7].

With the advent of the digital era, which has reshaped and introduced new forms of healthcare, the conveyance of this clinical empathy – the understanding of emotional states, communicating, and acting upon this – has been challenged. The growing presence of AI-operated tools and robotics, trained to mimic empathetic responses, illustrates this shift. In addition, new forms of digital communication, such as live chats (operated by humans or AI) in health-related settings, introduce unique dynamics to empathetic exchanges. This is because digital platforms change the way we interact, removing or altering traditional cues like tone of voice or facial expression. Therefore, understanding how clinical empathy can be effectively conveyed in digital, real-time textual mediums is pivotal. However, so far, research on digital clinical empathy for text-based, real-time communication tools such as live chats is limited, especially with regard to a differentiation of the three processes of empathy from the perspectives of both users and health professionals. As live chats become increasingly prevalent, the aim of this study was to explore how health professionals can effectively understand emotional states, communicate an understanding of them, and act upon this understanding, using written language in live chat services.

### Clinical empathy

Clinical empathy is characterized by a clear division of roles: the professional healthcare worker who empathizes and the patient who receives empathy [6]. Moreover, it has been defined as “a predominantly cognitive (rather than an affective or emotional) attribute (brain mechanism) that involves an understanding (rather than feeling) of the patient’s pain and suffering, combined with a capacity to communicate this understanding (behavioral component)” [8]. Thus, while empathy in non-professional, informal realms may involve experiencing the emotions of another person (or imagining them), clinical empathy focuses more on understanding and acknowledging these emotional states, communicating this understanding back to the patient and, acting upon this. Studies and reviews on clinical empathy list a series of techniques or elements to foster clinical empathy in relation to each of the processes of empathy [3, 9–12]. Hence, rather than a personality trait, clinical empathy is perceived as a skill that can be learned and improved, and its promotion forms an integral part of medical education and training [11, 13]. This is particularly relevant in situations where there is a scary diagnosis such as cancer and / or a particularly stressful phase of disease, which is often accompanied by considerable psychological stress [14].

In the area of recognizing and understanding emotional states, techniques include active listening, a method wherein health professionals strive not only to hear but comprehend the patient’s emotions and experiences; and perceiving, interpreting, and responding to both verbal and non-verbal emotional cues from the patient [3, 11]. The literature describes how to communicate understanding of emotional states, validation and recognition of responses, and authenticity in interactions [9–11]. Acting upon this understanding is context-dependent and can involve laypeople-oriented communication, especially when describing medical terms, or providing additional support or resources, e.g., psychological support [9, 15]. Clinical empathy also involves a balance between emotional engagement and detachment, which allows healthcare professionals to be compassionate, supportive, and understanding, while still being able to make rational medical decisions and avoiding emotional fatigue or burnout [3, 15].

### Digital clinical empathy

Shifting the focus from traditional modes of empathy, the digital realm requires a new arena of empathy research. Digital empathy, a prospering concept in the literature, encapsulates the understanding of emotional states and communicating and acting upon this understanding through digital media and technologies [16]. Digital empathy goes beyond face-to-face interactions, shedding light on how empathy requires to be recalibrated when technology comes into play. In this, technologies can either mediate empathy conveyance in human-to-human interactions, such as between two individuals over a video chat or written chat, or facilitate human-machine interaction, like when an AI chatbot is designed to decode and respond to human emotions. As new modes of digital technologies have reshaped communication between healthcare professionals and patients, it is necessary to explore empathy in these digital contexts – hence to investigate digital clinical empathy in its many facets [17]. A conceptual framework from Gronding et al [18] illustrates how communication mediums act as filters each of the processes of empathy is influenced by the medium’s richness, immediacy of feedback, transmission quality, and content. Even before the COVID-19 pandemic, telepsychiatry had already begun reshaping communication between healthcare professionals and patients and proven to be effective [19], necessitating an exploration of empathy in digital contexts. Studies prior to the pandemic, such as one from 2015 [20] highlighted that patients often felt a strong empathic connection during telemedicine consultations, potentially due to the more direct eye contact characteristic of these interactions compared to typical in-person consultations. The pandemic further accentuated this need to investigate digital clinical empathy. Research during this period showed that patients perceived therapists as equally or more empathetic in online sessions than in traditional settings. This suggests that healthcare providers adapted or developed new skills suited to digital media, like interpreting limited visual cues and maintaining ‘camera-eye’ contact [21–23] [21, 23]. Therefore, while emerging evidence suggests a nuanced adaptation of empathetic techniques in online psychotherapy, particularly via video conferencing, our understanding remains even more limited for other digital communication channels, e.g., text-based tools like live chats.

### Empathy in written live chats

Emerging digital healthcare interactions have taken the form of real-time live chats, an increasingly common feature in digital services, particularly for customer interactions in business contexts [24, 25]. Live chats are popular for their immediacy, personalization, availability during off-hours, and user control over the conversation pace [26–28]. These live chats can be powered by humans, or AI-operated tools and robotics or collaboratively operated by AI and supervised by human agents. In many business settings, AI chatbots are gradually taking over customer service roles, sometimes even supplanting human agents [25]. Given their accessibility and popularity, particularly among younger demographics, variously operated models of live chats have also been employed in various health-related arenas, such as mental health services [29], cancer advice [30–33], and for health promotion [34]. While AI chatbots have demonstrated reliable guidance for users in areas such as business or finance transactions, they have shown limitations in providing medical advice [35–37]. There have been instances where AI chatbots have given misleading advice or failed to articulate the rationale or source behind their otherwise medically correct recommendations [36, 38, 39]. This highlights the intricate complexities involved in healthcare advice, requiring nuanced understanding and human judgment, elements AI currently lacks.

Beyond the provision of correct, evidence-based information through live chats, it is equally critical to consider how information is conveyed, aligning with the emotional needs of users. This consideration foregrounds the relevance of digital clinical empathy in the realm of live chat interactions. In this, increasing interactions between humans and technology have led to the emergence of Emotion AI or affective computing [40, 41]. Emotion AI has shown increasing proficiency in decoding and recognizing human emotional states based on the analysis of facial expressions, tone of voice, or writing styles in recent years [36]. With the advancements of Emotion AI, distinguishing AI chatbots with humanlike features from human agents becomes increasingly difficult for users, especially when the interactions are brief, as an experiment with more than 1.5 million users showed [42]. In this experiment, users were randomly assigned to chat with either a human or AI counterpart for two minutes, followed by the user’s guess about the nature or their encounter [42]. Despite continuous advancements in decoding emotional states, AI is fundamentally incapable of experiencing emotions and constrained to mimic empathy. This imitation can, at times, lack the depth and authenticity of genuine empathetic responses. Moreover, the responses are often bound by a narrow frame of topics, and there is a risk of the user’s emotional state being misunderstood or not conveyed effectively [36, 41, 43, 44]. Such limitations in AI’s empathetic abilities have been found to hinder the acceptability of chatbots, especially in sensitive, emotionally-charged contexts [24, 45, 46]. Thus, while AI has immense potential in handling less complex, straightforward queries and analyzing emotional cues, its limitations are accentuated when faced with more complex medical questions. In the current state of technology, this provides a compelling argument for the necessity of human health professionals in providing medical information, particularly in the context of complex healthcare questions.

On the flip side, human health professionals face their own set of challenges in grasping and responding to patients’ perspectives and emotional states when using text-based digital tools [29, 47, 48]. Studies using text-based, written tools echoed challenges in both understanding perspectives and emotional states as well as communicating and acting upon this via chats [29, 47, 48]. This may be as written interactions are devoid of tonal and visual cues that play a role in understanding and communicating empathy in face-to-face interactions. Furthermore, these interactions are essentially disembodied, putting an onus on the written word to convey empathy effectively [5].

While healthcare providers are trained to justify their knowledge, apply ethical standards, and adopt clinical empathy in face-to-face encounters, there is a dearth of research focusing on digital clinical empathy for health professionals, particularly within the context of written live chats. As live chats become increasingly prevalent, understanding how to convey and adapt empathy to help-seeking users within it is critical. It is hence essential to explore how healthcare providers can effectively understand emotional states, communicate an understanding of them, and act upon this understanding, using written language in live chat services.

Familial cancer, which can have genetic and/or lifestyle-related causes, affects not only the patients but also their relatives and is associated with great anxiety and information needs [49], making it a good model topic to explore digital clinical empathy.

Therefore, our aim was to investigate the methods of digital clinical empathy in a human-to-human, written live chat, with a specific focus on understanding what users expect and desire from such interactions. Hence, we formulated the following research questions (RQs):

RQ1: What are expected techniques of digital clinical empathy in a human-to-human live chat from the perspective of potential users and responding health professionals with regards to:

a. understanding users’ perspectives and emotional states,

b. communicating this understanding,

c. and acting upon this understanding?

Utilizing these findings for training purposes, we applied and evaluated techniques of digital clinical empathy in actual chat encounters:

RQ2: What are implemented techniques of digital clinical empathy in a human-to-human live chat from the perspective of potential users and responding health professionals with regards to:

a. understanding users’ perspectives and emotional states,

b. communicating this understanding,

c. and acting upon this understanding?

## Methods

### Study design

This study is part of a larger collaborative project between two universities, a Cancer Information Service (CIS) – a government-funded institution that provides free, evidence-based information on cancer-related topics to the public–, and a patient organization for familial cancer, the BRCA-Network. This project accompanies the formative and process evaluation of a live chat service focused on the topic of familial cancer at the CIS [32, 33], comparable to a chat service implemented at the American Cancer Information Service [30]. The live chat is operated by health professionals and provide personalized, evidence-based information to users with questions relating to familial cancer, prevention and early detection. The CIS hosts the live chat service; the universities are primarily responsible for conducting qualitative and quantitative formative and summative evaluations of the service; and the patient organization contributes by providing advice and patient perspectives.

Given that cancer brings with it many uncertainties for the person involved, empathy has proven to be key to cancer-related communication [6] and thus, has been a focal point during the planning period of the live chat service. The current study focuses on investigating the expected techniques of digital clinical empathy for the chat and evaluating to which extent these were implemented in chat encounters, drawing on both perspectives: the users and the responding health professionals. It started with (1) focus group discussions with potential live chat users and interviews with CIS physicians (i.e., the health professionals) in October/November 2021, relating to RQ1. Questions centered around evaluating the appropriateness, effectiveness, and challenges of a live chat for cancer-related questions, with a focus on the role of empathy in digital written communication from the perspectives of potential users and health professionals (see further details in the supplementary file 1). Results from the focus group and interview study were shared with the CIS and used for training purposes with the chat team and development of the chat application. Before launching the chat for the public, we conducted (2) two rounds of usability tests in January and April 2023, addressing RQ2. In the usability tests for the live chat service, potential users and CIS health professionals interacted with the chat system as it would be experienced by end-users, i.e., simulating user inquiries and responses. Users joined the chat encounter while on video conference, sharing their screen and ‘thinking aloud’. The ‘think aloud’ methodology [50, 51] is commonly used for usability tests, where potential users verbalize their thoughts while using the new product or service. In our study, it was used to capture immediate user reactions of their experience using the chat. As empathy, especially in a digital context, can be nuanced and is grounded in the users’ perspectives, thinking aloud, which is conducted in real-time, enhances the authenticity of user responses compared to retrospective accounts [51, 52].

The usability tests were followed by interviews with the users and reflections with the responding CIS team members, focusing on the expression and perception of empathy in the live chat, exploring how empathetic communication is achieved and received in a written format, and evaluating the overall effectiveness of communication in addressing users’ concerns in a digital healthcare setting (see supplementary file 1 for question guides).

### Participants and procedures

#### Phase 1: interviews and focus groups

To gain insights into expectations and practice of digital clinical empathy for cancer-related inquiries, we recruited *n* = 7 CIS physicians, experienced in engaging with the public through different existing services (e.g., telephone and email service, social media, research management) for semi-structured interviews.

For the focus groups, social media and other digital posts shared by the CIS and cancer support groups were used to recruit *n* = 42 younger, digitally-oriented people, i.e. participants who had extensive experience using various digital written communication channels and who could be potential users. This demographic emphasis is strategic, considering that younger generations, who are typically more engaged with digital communication platforms and at risk of familial cancer due to genetic mutations, have been underrepresented in existing CIS services like telephone and email, which predominantly attract an older demographic (aged 50 and above) (see [33] for further details about recruitment strategies). Of those, *n* = 11 focus group participants had no prior experience with cancer, and *n* = 31 participants carried a genetic disposition associated with increased cancer risk and/or had experienced a cancer diagnosis. Focus group participants were divided into seven groups with five to seven participants, separating those with and without cancer/mutation experience.

Following the procurement of informed consent, both interviews and focus group discussions were conducted with the online conference tool Zoom (Zoom Video Communications, Inc). Sessions were guided by semi-structured interview and discussion guidelines in line with RQ1 and spanned durations between 21 and 55 min for interviews and 52 and 95 min for focus groups. Interviewees were not provided with monetary compensation. Participants in the focus groups were compensated 25 € for their involvement. All interviews and focus group discussions were facilitated by the lead author, who possesses extensive expertise in collecting qualitative data and was supported by student assistants. Data were gathered in German and quotations used within this paper have been translated accordingly. Interviews and focus groups were audio-recorded, transcribed, and fully anonymized.

#### Phase 2: usability tests

Two rounds of usability tests with ‘think aloud’ methodology were held in January and April 2023. Participants in the usability tests consisted of *n* = 9 health professionals who operated as CIS chat respondents (eight physicians, one lawyer with a specialization in medical social legislation) and participated in both rounds; and two different sets of users (*n* = 18), who participated in the think aloud usability tests. One CIS chat respondent had participated in the interviews of the first phase of data collection. Users of the first round of usability tests had participated in the focus group discussions, the second set were newly recruited participants with no prior experience with our project who responded to social media posts by the CIS and different cancer support groups.

For the usability tests, the CIS health professionals operated the chat through the CIS’ website integrated chat platform embedded within their website. The first author and a research assistant conducted video conferences via Zoom with the users, who shared their screens throughout the chat encounter. Users were encouraged to ‘think aloud,’ articulating their thoughts and reactions verbally by talking during the written chat. If participants remained silent, the first author engaged them with prompts, such as asking them to describe their current thoughts. The users’ comments were documented in detail in a structured protocol by a trained research assistant. This protocol was pre-categorized into sections like positive/negative feedback relating to technical features, communication etc. Additional data, including chat topics, duration, and the number of questions asked, were recorded post-chat using the chat transcripts. Following the chat, each user was interviewed about their chat experience with a focus on empathy in communication. The interviews were audio-recorded and an anonymized, detailed transcript of the interview with selected quotes was prepared in conjunction with the think aloud protocol. Following both rounds of usability tests, CIS health professionals in their role as chat respondents were asked for a written reflection on their chat experiences, guided by a set of main and sub-questions (i.e., strategies of identifying the emotional state, mood of the users, and responding to those). Eight of the nine CIS health professionals handed in their reflections on a digital sheet. The participant characteristics for each of the samples are summarized in Tables 1 and 2.

**Table 1:** Sociodemographic data of phase 1 and 2 users

Characteristic	Phase 1 (focus group)		Phase 2 (usability test)	
	n	%	n	%
**Cancer experience**				
Cancer experience or genetic disposition	31	74	13	72
No cancer experience	11	26	5	28
**Gender**				
Female	28	67	14	78
Male	14	33	4	22
**Age**				
18–29	13	31	5	28
30–39	17	41	12	67
40–49	6	14	1	5
50 +	6	14	0	0
**Education**				
Academic degree	27	64	15	83
Non-academic degree	15	36	3	17
**Migration background/Non-German native**				
Yes	5	12	2	11
No	37	88	16	89

**Table 2:** Professional backgrounds of phase 1 and 2 health professionals

Characteristic	Phase 1 (focus group)		Phase 2 (usability test)*	
	n	%	n	%
**Professional background**				
Physician	9	100	8	89
Lawyer	0	0	1	11
**Position within the CIS**				
Telephone service	3	34	4	45
Email service	3	34	4	45
Social media	1	10	0	0
Research management	2	22	1	10
Chat service	0	0	9	100

* Note: Health professionals were not solely members of the chat team and partially also worked in one other CIS service

### Data analysis

Each of the data sets for the two phases of data collection was subjected to an initial independent analysis, utilizing qualitative content analysis [53]. An introductory coding frame was constructed in accordance with the focal themes for each phase (deductive procedure) and new subcategories were formed, derived directly from the data (inductive procedure). For instance, under the deductive code ‘understanding/expressing need’ from the perspective of a potential user, we identified inductive codes such as ‘not knowing how to start/express feelings’ and ‘different means to express emotions in chat’. Additionally, the same pre-selected code, when viewed from the health professional’s perspective, revealed emerging codes like the ability to read ‘nuances’, the importance of ‘not interpreting’ prematurely, and the need to ‘figure out emotional state throughout chat’.

Following this step, axial coding was performed, rooted in Grounded Theory [54], whereby subcategories underwent a review process and were linked, collated, and refined to ensure their mutual exclusivity. A thorough re-examination of all transcripts, coded portions, and the final coding frames was undertaken before the different perspectives for each phase were joined and compared. Distinguishing between the processes of ‘b. communicating understanding’ and ‘c. acting upon understanding’ deserved particular attention in this text-based environment, where actions are also a form of communication. In our analytical approach, ‘communicating this understanding’ encompasses codes that highlight communicative acts to acknowledge or validate expressed emotional states or perspectives. On the other hand, ‘acting upon this understanding’ is defined by coding communicative acts that go beyond mere acknowledgment—these acts could involve the provision of additional resources, initiating a referral, or taking steps to address emotional needs directly. Thus, the difference lies in the intent and impact: the former focuses on expressing comprehension and validation, while the latter takes concrete steps based on that understanding.

The data from phase 1 (focus groups and interviews) were amalgamated in response to RQ1, while the data from phase 2 (usability tests) were used to address RQ2. The coding frames of both phases were compared. The first author and two student assistants performed the coding of the data. To ensure the validity and reliability of our coding scheme, a rigorous process was followed. The first author began coding with five transcripts from each dataset, developing the initial categories. Subsequently, student assistants, one for each phase, were trained to use this coding frame. To assess intercoder reliability, two transcripts from each dataset were coded independently by both the first author and the student assistants. Discrepancies in coding were discussed and resolved, leading to further refinement of the coding frame. The first author reviewed all coded transcripts by the research assistants. The MAXQDA software (VERBI GmbH) was utilized to facilitate computer-assisted data analysis.

### Ethics approval and consent to participate

The research project was granted ethical clearance by the ethical board of Bielefeld University. Prospective participants from all samples received an information sheet, detailing their role, rights, type of data collection, data protection, and a consent form. Informed consent was obtained from all research participants.

## Results

### Overview

The results section is divided into two sections in line with RQ1 and RQ2. Each of these two main sections has three subsections in line with the three-fold processes of empathy, contrasting the perspectives of (potential) users and health professionals (see participant data in methods section). Each identified theme is marked in italic and accompanied by illustrative quotes. A summary is provided at the end of each subsection and the results conclude with a summarized table that encapsulates the key findings and interpretations across all phases, offering a cohesive and comprehensive overview of our results.

To distinguish participants from the different samples, the following terminology will be applied (Table 3): ‘Participants’ includes perspectives from both samples during one phase of data collection. CIS health professionals from the first phase are referred to as ‘CIS interviewee’. As all CIS interviewees are women, their gender is not stated. Focus group participants (i.e., the potential users) are referred to as FGP followed by their gender (F for female and M for male) and age, e.g., ‘FGP M21’. Usability test users (UTU; i.e., the users) are distinguished by rounds 1 and 2 (R1 and R2) as well as their gender and age (e.g., UTU R1 M21). The CIS chat respondents are referred to as ‘CIS respondent’, again without gender as all are females.

**Table 3:** Overview of used terminology for different samples and research phases

Phase	Participants	
	Potential Users	CIS health professionals
1 (Focus groups, interviews)	FGP M/F Age	CIS interviewee
2 (Usability tests)	UTU R1/R2 M/F Age	CIS respondent

### Expected techniques to foster digital clinical empathy (RQ1)

#### Understanding users’ perspectives and emotional states

In the exploration of digital clinical empathy within the context of human-to-human live chat interactions, the first process of empathy to consider is the understanding of the user’s perspective and emotional state.

Participants from both samples (FGP and CIS interviewees) believed that understanding and recognizing the (emotional) needs of the user was key to enacting empathy, yet prone to interpretation errors in a chat. Several FGPs described it as a *challenge to express needs* when faced with a diagnosis of cancer (or its probability):

“If you have received a diagnosis, you may not know how to express it. So that means that the person who has to answer in the chat would have to be able to give a good, reflected answer based on relatively unspecific statements.” FGP F21.

“Writing has no tone and nuance. When it comes to the advice seekers, it’s just incredibly difficult in a chat to express the emotions one has when dealing with cancer. And for the advisors, it will be difficult to see this and to calm people down or to discern if the advice seeker just wants objective information. Finding that out is very difficult if you only have text.” FGP F39.

There were also some views that emotional states could be deciphered in chat encounters due to *variations in expressions* as one FGP, who worked in customer chat service, explained:

“From my own experience, I can say that the people who turn a live chat write as differently as they are. Some use emojis, others comment statements with “haha” and others have fears and concerns and you will also read this from the way they write. We all know from WhatsApp and other messengers within our private networks that people have very distinct ways to write. The chat team needs to look at everyone who comes into the live chat individually and respond as you would do in an oral conversation.” FGP F31.

Similarly, some FGPs viewed mutual engagement and *explicitness of their needs* as an element to foster understanding of their emotional state:

“If I want to be understood, it also comes with a responsibility from my side. Like in the real world, I mean non-digital encounters, I can’t expect my counterpart to understand me right away. I need to open up about my feelings and even more so if we are just texting. However, not everyone is capable to do so or knows how to express themselves. So I still view it as a delicate matter to find the right tone in a live chat on cancer.” FGP M30.

Views from the perspective of CIS interviewees were also mixed wherein half of the participants described presumed challenges in recognizing how the user felt. Others described *understanding user needs as a process* of mutual engagement throughout the chat:

“You might not know right from the start who and in which kind of state is chatting with you, but I believe by being open and friendly, you give users the opportunity to open up and you’ll know how to engage with them over the course of your encounter.” CIS Interviewee.

In summary, understanding a user’s perspective and emotional state proves multifaceted and mutual, encompassing both user’s expression of needs and respondent’s interpretation. The diverse perspectives within the FGP and interview samples underscore the dynamic nature of the interplay of this empathetic process. While difficulties in expressing needs in an emotionally charged situation such as a cancer diagnosis was described as a challenge, different writing styles and recognizing needs in the process of interacting were described as strategies to gain an understanding of the user’s needs.

#### Communicating this understanding

Following the recognition of the user’s needs, communicating this understanding back to the recipient is the second facet of empathy.

Participants in the study detailed the various elements involved in conveying this understanding within the context of a live chat setting. From the user’s perspective, the majority of FGP participants highlighted the value of a *timely response*, indicating that a swift reply is considered an essential part of making them aware their need had been received.

“When chatting, you just don’t have eye contact and you don’t see or feel that now really someone is waiting. Especially when it comes to feelings or the like, then you may not feel so left alone if you get a quick response.” FGP M30.

“I have that expectation that I get a prompt feedback. What promptly means – I can’t say right now, but at least an in-between message that they [chat respondents] have received my message, that it has been seen and that they care about it.” FGP F35.

Moreover, many FGPs emphasized the importance of *genuine engagement*, which includes a personal address by their chosen name in the chat and avoiding formulaic or empty phrases. There was also agreement that respondents should refrain from attempting to take their perspectives:

“If they would write something along the lines ‘I am sorry for you and your family’ that would rather trigger me. As a cancer patient, you get enough compassion from your immediate social network. Here [in the chat] pity comes across strangely and seems like a phrase even if it is perhaps well-intentioned.” FGP F32.

“People sometimes say that they can imagine how difficult my situation is. But honestly, hardly anyone can. That’s why I would avoid any such wording.” FGP F34.

From the perspective of FGPs, there were contrasting views on how *the user’s worries should be handled*. Some FGPs expressed a desire for respondents to avoid language that could potentially exacerbate fears, while a few felt “seen” and taken seriously in having their worries acknowledged.

“Let’s imagine I enter the chat because I suspect my cancer has come back and I am very worried. If the person in the chat would now acknowledge that it was right to be worried, my thoughts would start spinning and I would most likely hear all they write with that voice in my head. So, I would much prefer a more positive connotation in the communication and that they are there to help me.” FGP M28.

“Having cancer means going through a lot. And sometimes it’s just nice if somebody sees and acknowledges this.” FGP F32.

FGPs also highlighted the need for *authenticity* in these interactions. This concept involved the respondent being forthright about their limitations, such as not having all the answers or not being fully acquainted with the user’s personal experience as the following quote shows:

“I want to know if my question can’t be answered. I think that makes a live chat very credible and also very authentic when I notice that the person sitting behind it is really someone who is not all-knowing.” FGP F31.

Being authentic and acknowledging limitations was also viewed as important by CIS interviewees, however, this could also evoke frustrations and not be understood as empathy as one participant, working in the CIS telephone service, explained:

“We can only listen and give advice, but we don’t perform examinations, and this limits us in what we recommend. But this is what people often want: a specific answer, a personalized recommendation. We have to get this across, but it is difficult and requires sensitivity.” CIS interviewee.

Some FGPs underscored the importance of *adaptive communication*, describing a preference for respondents to adopt a more formal or informal communication style along with the user’s communication style:

“I would dislike a very formal approach, it broadens the distance in this chat even more.… Matching my style, this is what I would appreciate.” FGP M27.

“I am talking to doctors and whether that’s in a chat or in real life, I don’t address them by their first name. Equally, I also expect them to address me formally.” FGP F35.

In contrast, all CIS interviewees viewed a more formal, *neutral communication* style as the better option to avoid miscommunication:

“If we take the extreme of informal – emojis. Do I hurt them when I want to cheer them up and send them a smiley face or a sun or something nice? It’s possible that it comes across all wrong, when their world is falling apart. So I would be very cautious with anything too informal.” CIS interviewee.

From the respondent’s viewpoint, CIS interviewees also detailed the importance of *in-depth questioning*, seeing it as a method to gain a complete understanding of the user’s (emotional) needs. They also pointed out the need to contextualize this practice:

“To give the user what they need, often a lot of questions and clarifications are needed. Sometimes, it is not clear to the users why we need all that information. To avoid feelings of ‘being interrogated’, it is helpful to elaborate on why these questions are necessary.” CIS interviewee.

In summary, the process of communicating an understanding of a user’s perspective and emotional state, as a facet of digital clinical empathy, revealed a complex interplay between the users’ and the respondents’ perspectives. Both the FGP and the CIS interviewees shared views about key elements like timely response, genuine engagement and authenticity. Divergent views, however, were observed on issues such as handling the user’s worries, with a contrasting need for both avoiding fear-evoking language and acknowledging existing fears. Similarly, while users preferred a more informal or adaptive communication style, respondents favored maintaining a neutral and professional tone.

#### Acting upon this understanding

Recognizing a user’s needs and communicating that understanding is followed by acting upon that understanding. In a live chat environment, the intended actions proposed by both users and respondents are diverse and multi-faceted.

For FGP participants, representing potential users, several elements were described as key to manifest empathy in a digital setting. An expectation revolved around *time commitment*. Most FGPs emphasized the importance of the responding physicians dedicating sufficient time to provide individual, personalized responses, ensuring that their needs (both emotional and beyond) are thoroughly addressed.

“Showing empathy in a traditional sense is a balancing act in this chat because of all the emotional nuances that are missing on both sides. To me, dedicating time to my inquiry and being outspoken about it – ‘You can ask me any question. Or what do you need right now?’ – is also a form of empathy to me.” FGP F38.

The importance of dedicating time was equally shared by the CIS interviewees and viewed as a technique to enact empathy:

“In all our services, we aim to convey that we have time and that we take the time to respond individually to the users. In the telephone service, we sometimes offer to call people back if they want to compose themselves first.… We would try a similar approach in the chat.” CIS interviewee.

An additional element of empathy mentioned by multiple FGP participants was the *provision of understandable information*, often mentioned in conjunction with the previous element of time commitment. Many FGP participants expressed the need for clarification of complicated medical terminologies or study results, providing a level of understanding often missed during consultations with their primary physicians.

“I’ve had that situation several times when I sat at home with my doctor’s letter after a visit to the hospital. And I either thought I had asked all the questions or I was just too overwhelmed to ask any more. And then you sit at home and stumble over words. And you ask yourself ‘How bad is it?’ and the worst thing you can do now is to start googling. If you had access to that chat or any other service with verified information and people who give you context and explanations – maybe I would not go bananas.” FGP M41.

In parallel, CIS interviewees also discussed the provision of individual, evidence-based information as a critical feature of their service. In this, several interviewees agreed it was important to *frame factual information empathetically*, find the “right tone” when conveying information so as to be perceived as empathetic:

“The information should not just be factually correct, but also humane and interpersonal, well understandable. And, as I said, I have the experience that people in fear filter every piece of information differently than someone else does. Information goes through a grid where everything bad is amplified and all fears are magnified. And it is important that you manage to convey information in the way you want to or you have the reverse effect.” CIS interviewee.

“You have to wrap your information so that your evidence becomes empathetic, personal and meets the user at the level where they are.” CIS interviewee.

Some focus group participants described *boundary recognition* by the respondents as another element of enacting empathy. This aspect covered the acceptance of a participant’s preferred level of anonymity and respect for the withholding of certain information.

“The nice thing about the tool is that it is semi-anonymous. It is a limitation, but also an advantage of the chat, because I’ve noticed that men, in particular, are often ashamed. They don’t want to talk about everything and disclose all their intimate details. If the chat respects that people take time to open up… it can become a safe space to address sensitive topics.” FGP M40.

This anonymity was viewed as a challenge by CIS interviewees who required a certain level of information in order to provide personalized advice, as several interviewees described:

“I think the chat might appeal to people who want to maintain a certain level of anonymity and I do understand that. What some people don’t understand is that the quality of the information we can provide varies with the amount of details we have.… As the respondent, I have to be clear why I need further information to make the user understand.” CIS interviewee.

Moreover, the CIS interviewees proposed *empowering users* to take further steps in their cancer journey and enabling informed decisions as acts of empathy the CIS aimed to enact to all their users, notwithstanding the communication channel they chose. An advisor working in the telephone service described this as follows:

“I have had telephone sessions with patients who were not well informed and only saw one option for themselves. This can go in both directions: those who overreact and want to be overtreated and others who don’t or don’t want to see the seriousness of their situation. And of course I am not their oncologist and I reach the limits of my knowledge which I have to admit. But I can do my best to support these patients in getting a second opinion or to think once more about their choices.” CIS interviewee.

Lastly, CIS interviewees articulated their intent to *‘go the extra mile’* and go beyond merely answering the users’ questions and thereby expressing empathy. This included potential referrals to support groups, hospitals, or other services within the CIS.

“We have the capacity to go the extra mile and offer users specific information beyond what they ask. If someone comes with questions about hereditary cancer, I can give them the addresses of specialized hospitals in their area, but also contact details of a support group or information about social services. I think it’s good to have a ‘customer first’ concept embedded in the service.” CIS interviewee.

In summary, acting upon a recognized need in a live chat requires several techniques, per the perspectives of both potential users and CIS interviewees. These include dedicating adequate time to individual inquiries, explaining complex medical content in an understandable manner, and acknowledging and respecting users’ boundaries. For CIS interviewees, extending help beyond the immediate scope of questions, facilitating informed decision-making, and careful phrasing of information were seen as vital techniques of empathy.

### Implemented techniques fostering digital clinical empathy (RQ2)

#### Understanding users’ perspectives and emotional states

During the second phase of data collection, understanding emotional states presented distinct nuances for both users and health professionals involved in the usability test.

Most users exhibited a heightened *awareness of the artificial setup* of the usability test. They entered the chat without the emotional baggage they might have brought into a real consultation, recognizing that the situation was a constructed setup:

“I entered the chat with a neutral feeling and I am also exiting it that way. I knew this was a test and didn’t come, let’s say, feeling desperate.” UTU R1 F34.

Health professionals also noted this observation, while also reporting *heterogeneous understandings* of users’ emotional states. For the most part, they felt confident about their grasp of the users’ emotions and needs, drawing from explicit indicators like emoticons and expressions of gratitude.

“I had the impression that I got the users’ needs. In the end, it’s like in a personal conversation: You need to engage with the user individually.” CIS respondent.

However, some health professionals expressed concerns and questioned whether their interpretations were accurate, given the lack of non-verbal cues that would ordinarily help interpretation in other settings.

“Compared to the telephone service, it turned out to be more demanding - due to the omission of other cues. Listening allows us to sense the caller’s emotions very well when the voice wavers, heavy breathing occurs or unusual pauses occur. In general, identifying what the user needed, also emotionally, remained unclear for longer or sometimes changed very abruptly in the course of the conversation, which may also be due to the ‘test situation’.” CIS respondent.

Simultaneously, some health professionals experienced *dual attention stress*, needing to focus on both the factual aspects of the users’ inquiries and their emotional needs. This dual attention demanded a heightened degree of focus and comprehension, augmenting the challenge of empathetically understanding users in the live chat setting.

In summary, the second phase showed some of the complexities of comprehending emotional states in a live chat setting. While the perceived artificiality of the chat test environment limited the full exploration of emotional needs of the users, health professionals had mixed impressions about getting the users’ needs right. They also experienced a dual challenge of addressing both factual inquiries and emotional needs.

#### Communicating this understanding

Several aspects stand out regarding the communication of understanding emotional needs during the second phase of data collection.

During the first phase, key elements such as genuine engagement, and authenticity emerged as shared views among both focus group and CIS interviewee participants. The second phase reinforced these findings, particularly with many users expressing appreciation for *timely responses* from the health professionals:

“I was quite impressed with the pace of the chat. The physician always responded swiftly, even if it was only to ensure she had understood me correctly.… I feel this was quite empathetic.” UTU R2 F42.

Responding timely to a user’s message was described as an employed technique by the CIS respondents and served different purposes (e.g., rephrasing the query, letting the user know they were searching for information). Another reoccurring theme was *in-depth questioning* as a technique to find out the user’s need and communicate this understanding back. Health professionals reiterated the use of questions to explore the user’s condition or concerns thoroughly. This involved seeking the user’s permission to delve into their medical or personal history. Occasionally, CIS respondents would also explain their rationale behind this questioning, as participants from both samples mentioned. Users overall valued this technique, demonstrating the health professional’s interest in their situation.

“I had the impression that I was in good hands with the person answering my questions; I noticed this from the doctor’s counter-questions. They were personal and directed at me.” UTU R1 F36.

Some observed, however, that it could make the chat feel protracted and difficult to scroll through the chat conversation.

“I got lost at some point and tried to scroll back to the initial question. While I can understand why they have to ask many questions, you have to bear in mind the technology and in this case, a small chat window, and navigating in between questions became complicated.” UTU R1 M30.

*Authenticity*, a pivotal element from the first phase, was further dissected in the second phase in terms of *writing styles*. The emergence of typos stood out in several post-usability interviews with users. While some users found typos “humanizing”, signaling the presence of a real person on the other side of the chat, most perceived them as less professional or potentially challenging for specific user groups, such as dyslexics or non-native speakers.

“I noticed the typos. At the beginning, I found them likable, but at some point, it became too much for me. Even though it didn’t affect my reading comprehension, I noticed that I perceived the person as less professional.” UTU R1 F34.

“As a non-native speaker, I had put some pressure on myself to spell everything correctly so that the health professional would understand me. Then I saw they also made mistakes. My German is very good and I didn’t have any issues understanding, but for someone else, there might be. I would prefer they check the message for typos before sending it.” UTU R2 F32.

Health professionals, on the counterpart, were aware of this and described this as *trade-offs* between the necessity of responding quickly and laypeople-oriented versus the potential for introducing errors:

“Correcting typos is time-consuming and slows down the writing process. A spell checker would be helpful here, but as far as I know, there is a problem with data protection. So for the time being, it remains a matter of weighing up what is more important: a perfect answer or a natural conversation that can have spelling mistakes.” CIS respondent.

“I increasingly tried to formulate the responses myself and copy less from our data bank. In my experience, this makes the communication faster and more empathetic, but it also carries a higher risk of accidental misinformation or typos.” CIS respondent.

This is in line with the previously identified divergence in preferences for communication styles which surfaced again in phase two. In contrast to expected adaptive communication by focus group participants, users and health professionals of phase two universally acknowledged a neutral, *formal communication* style during the usability tests. These had a varying reception among the users of whom some valued professionalism and others expressed a preference for a more personalized and less distanced interaction.

“I felt self-conscious at the start as I was addressed so formally and I saw the doctor title [of the respondent]. I wrote and rewrote my question several times, trying to formulate an eloquent question.” UTU R1 M29.

“The writing style was very pleasant for me. Since the topic is emotionally charged, it is pleasant to write with someone in a factual manner.” UTU R1 F37.

In summary, while users appreciated the technique of timely responses and tolerated in-depth inquiries, they held mixed views on the formal language and typographical errors in the chat. On the other hand, health professionals described how they tried to balance swift, accurate responses with maintaining a fast, authentic and approachable writing style that translated complex medical language from databanks into simpler, user-friendly expressions, a technique that was positively received by users.

#### Acting upon this understanding

This section explores the third process of empathy in the usability test – acting upon an understood need of the user. During the second phase of data collection, several themes emerged that were in alignment with the first phase’s expectations.

Time commitment was universally appreciated by the users who felt that the health professionals took the time necessary for each consultation, reinforcing the importance of dedicating adequate time to individual inquiries.

“She asked, ‘Do you have any further questions or would you like to end the chat?’ and then I could have said, ‘No, I have another question’. So she left it up to me to decide and I liked that very much.” UTU R1 F49.

From the health professionals’ perspectives, this technique aimed to create a user-centric communication where users could decide the duration and pace of the chat. Nonetheless, some health professionals questioned how much time they could dedicate per user once the chat was launched:

“Inasmuch as we want to dedicate as much time to every user as they need, we also have to view it from a practical perspective. Once the chat is launched, there might be queues. How much time can and should we take for every chat?” CIS respondent.

Emphasized from phase one and slightly nuanced during the usability tests, *providing valuable information* in the chat was described a relevant enactment of empathy. This encompassed sharing relevant, recent studies and guiding users through the next steps of their care journey. Nonetheless, constraints with the chat format were acknowledged by some users who expressed dissatisfaction when their questions were either addressed superficially or deemed too intricate for the chat’s capabilities. While these users acknowledged the inherent limitations of a chat service, their experience was marked by a degree of frustration when their expectations were unmet:

“Well, you should bear in mind that this is a chat and not suitable to ask complex questions. I wished I had received a more specific answer to my question. Maybe I could have known this before.… I feel a bit weary.” UTU R1 M21.

CIS respondents were also aware that some questions or issues might not be adequately answered in a chat. The offer of *referrals* to services inside and outside the CIS was viewed positively overall by users, though some found referrals to other CIS services unnecessary, if, for instance, their question had been sufficiently answered or they had chosen the chat for a particular reason.

“The suggestion of talking to the telephone service came out of the blue for me because I felt the question had already been answered. So that made me wonder if there was more to know.” UTU R1 F34.

“I would turn to the chat mainly to stay anonymous and when I go to the website, I can see that I can also make a call and if I choose the chat, they [respondents] should make it clear why they suggest a phone call to me.” UTU R2 F33.

For some of the health professionals, the challenge was not only identifying the need for referrals but also *packaging referrals* in a way that would not make users feel dismissed.

“How and when to suggest referrals so as to not make users feel we ‘are brushing them off’ was a topic that we discussed in our team. In my usability tests, I suggested one referral and I think I found a good way to present it.” CIS respondent.

Finally, many users highly appreciated the reassurance from health professionals that they could return to the chat anytime. This *encouragement for future engagement* aligns closely with the first phase’s emphasis on fostering an environment that empowers users and goes beyond what is asked, uttered by both users and health professionals:

“Dr [X] invited me to ask any further questions now or at some point in the future and I really appreciated this. It’s a small thing to say but it gives me a positive feeling.” UTU R1 F35.

In summary, the third process of empathy, acting upon an understood need, incorporated a variety of techniques consistent with expectations from phase one in the usability test. Users and health professionals alike emphasized the importance of time commitment in chat encounters, creating an environment where users could control the duration and pace of the encounter. Information management was critical, with users appreciating the relevance and understandability of the provided information, despite some instances of perceived superficiality or complexity. The theme of referrals emerged as a nuanced area, with users generally viewing them positively but questioning their necessity in certain contexts. For health professionals, the challenge lay in framing these referrals in a non-dismissive manner. Encouraging future engagement was unanimously well-received, reinforcing the initial emphasis on creating an inviting environment for ongoing assistance.

In line with the three-fold process of empathy, both phases and their themes are summarized in Table 4.

**Table 4:** Overview of expected and implemented digital clinical empathy techniques

	Expected techniques		Implemented techniques	
	Potential users	CIS Health professionals	Potential users	CIS Health professionals
Understanding	- Challenge in expressing needs - Variations in expressions - Explicitness of needs	- Understanding needs as a process	- Awareness of artificial setup	- Heterogeneous understandings - Dual attention stress
Communicating this understanding	- Timely response - Genuine engagement - Handling worries - Adaptive communication	- In-depth questioning - Neutral communication	- Timely responses - In-depth questioning - Formal communication	
	- Authenticity		- Authentic writing styles	- Trade-off between quick responses and making errors
Acting upon this understanding	- Time commitment		- Time commitment	
	- Provision of understandable information - Boundary recognition	- Framing factual information empathetically - Empowering users - Going the extra mile	- Provision of valuable information - Referrals	- Packaging referrals-
			- Encouraging future engagement	

## Discussion

### Study summary

This qualitative, multi-method study analyzed which techniques of digital clinical empathy, a crucial element in patient-centered care, are expected and evaluated in the digital communication format ‘live chat’ on familial cancer in healthcare. While clinical empathy in face-to-face interactions employs techniques such as active listening, and a balance between emotional engagement and detachment [3, 11], the digital era has introduced new dynamics to empathy which need to be explored in the context of the technology [21, 22]. Given the growing prevalence of written live chats and the need to understand how healthcare providers can effectively convey empathy in this medium, this study addressed digital clinical empathy. Focusing on the three-fold processes of empathy – understanding emotional states, communicating, and acting upon this understanding – we investigated the expectations, implementation, and evaluations of techniques to foster empathy in a cancer live chat service at a Cancer Information Service (CIS). Our data combined the perspectives of potential users and health professionals during two phases: in focus group discussions and interviews before and in usability tests, followed by written reflections after the live chat encounter.

### Principal findings

During the first phase of data collection (focus groups with potential users and interviews with CIS health professionals), hence focusing on the required features of digital clinical empathy, the complex nature of empathy in digital environments became evident. As highlighted by both the potential users and health professionals, understanding emotional states was viewed as a reciprocal process that demands engagement from both parties. This is different from the process of clinical empathy in non-digital settings where understanding emotional states relies heavily on active listening of the health professional [3]. Moreover, unique to the live chat setting, the respondents have to rely on written cues and interactive engagement to infer emotional states, as they lack access to the visual and tonal cues typically available in face-to-face interactions.

In terms of communicating this understanding, the importance of timely responses, authenticity, and genuine engagement was reiterated across both samples, reflecting similar principles of clinical empathy in traditional settings [9, 10]. However, the study found diverging views on the nature of communication. The CIS health professionals, reflecting the professional orientation of clinical empathy, favored maintaining a neutral and professional tone. Meanwhile, the users preferred a more adaptive, and at times informal, communication style while not using empty phrases. This finding underlines the need to recalibrate the ways in which empathy is communicated in live chat settings, balancing professionalism with the informality often associated with digital communication [55].

Regarding acting upon an understood emotional state and need, both samples agreed on the importance of dedicating adequate time, making complex medical information understandable, and respecting users’ boundaries. These elements reflect practices associated with clinical empathy, such as clear communication and a patient-centered approach [8, 15]. Unique to the live chat environment, CIS health professionals emphasized the need to go beyond immediate inquiries and facilitate informed decision-making. This suggests that in the context of live chats, ‘acting’ might encompass not only addressing the immediate concerns of the user but also proactively offering relevant information and further support.

During the second phase of data collection (usability tests with interviews and written reflections), users and health professionals reflected on their experiences of empathetic communication in the usability test setting. This served as a reality check against the required features and expectations from the first phase. The second phase expanded and challenged some findings from phase one. While understanding users’ emotional states was confirmed as complex, the artificiality of the usability test and the intertwined nature of factual and emotional needs amplified this complexity. This underscored the need for a nuanced approach tailored to digital communication, especially when dealing with sensitive health topics such as familial cancer [31, 56].

The usability tests also offered additional insights on communicating a recognized need as health professionals described balancing swift, authentic responses while maintaining an approachable writing style. Users’ reactions to language formality and typographical errors were mixed. This highlights a tension between the desire for immediate interaction, inherent in the chat format, and the need to maintain accurate and reliable information exchange [57].

Acting on an understood need involved unique challenges and opportunities in the digital written context. Health professionals’ time commitment was highly valued by users, yet the live chat format also allowed users a new level of control over the pace and duration of the consultation. Providing valuable information in a chat-suitable format, however, surfaced as a unique challenge with users indicating some responses as too superficial or complex for the chat format.

Referrals, a commonly used strategy in traditional settings, presented new nuances in the digital context. While generally well received, some users questioned their necessity in specific chat contexts, while health professionals highlighted the need for careful framing to avoid users feeling dismissed.

### Implications for theory, methods and practice

In terms of theoretical implications, this study shows the necessity of adapting traditional understandings of empathy, largely derived from face-to-face interactions, to suit digital platforms such as live chats [16]. Here, our study highlights the value of distinguishing between the three processes of empathy [2, 3] and describes different techniques for each of them. This nuanced perspective can provide a more comprehensive understanding of empathy in both digital and non-digital healthcare settings. However, the study also points to the complexities in delineating these processes, as they often occur concurrently and interactively, suggesting that empathy may function more as a dynamic, interwoven process rather than isolated stages [58].

As live chats are increasingly operated by AI bots and robotics, our results underscore the intricate skills required for truly empathetic digital interactions. While current AI has progressed in identifying emotional states and partially in reflecting that understanding back to the user, the multifaceted task of empathetic responses – or acting upon this understanding – remains a challenge in AI development [40, 44, 45]. This challenge, however, presents a direction for future research and may open an avenue for a hybrid, cooperative model that harnesses the strengths of both humans and AI. This way, AI’s precision and speed in recognizing emotional states, especially in difficult areas as solely text-based interactions, can be integrated with the nuanced understanding and empathetic response of human health professionals.

Our study’s methodological approach, integrating the perspectives of both (potential) users and (responding) health professionals before and after the live chat’s development, offers valuable insights. This methodological approach underscores the importance of integrating user and professional perspectives right from the design phase, rather than retrospectively adjusting strategies and techniques. Moreover, the subsequent testing and evaluation phase further strengthens the research and implementation process, allowing for adjustments based on feedback and reflections. This emphasizes the importance of continuous testing and adaptation in the face of evolving user needs and technological advancements [59].

From a practical perspective, healthcare providers navigating digital spaces must find a way to uphold professional standards while exhibiting authenticity and conveying empathy [21, 60, 61]. This requires an understanding of different techniques, adapted to the specific digital medium [5]. Acknowledging this, our study results offer recommendations for healthcare professionals, summarized in Table 5. These recommendations, derived from our findings, could enrich professional training, enhancing digital patient interaction skills.

**Table 5:** Techniques for digital clinical empathy in written environments

Technique	Description
Engage genuinely	Ensure genuine engagement, particularly by addressing the user by their chosen name and avoiding formulaic responses
Respond timely	Prioritize swift replies to convey attentiveness and acknowledgment of the user’s concerns
Recognize and respect boundaries	Acknowledge the user’s preferred level of anonymity and respect their choice to withhold certain information
Be transparent	If in-depth questions or further research are necessary, explain the reason behind longer waiting times or many questions
Acknowledge user’s emotions	Recognize and communicate back the user’s feelings and emotions
Utilize adaptive communication techniques	Be flexible in communication style, adapting to the user’s tone and preference
Provide proactive support	Recommend or suggest additional services, anticipating patient needs beyond their immediate inquiries
Encourage future engagement	Invite users to return for further assistance or questions in the future, fostering a sense of ongoing support and accessibility

Finally, the challenge of balancing authenticity and accuracy in a live chat setting points towards the need for additional support and quality control measures in digital healthcare, e.g., complemented by AI or other automated tools. Ensuring the accuracy of information disseminated in such platforms is key to being a reliable, user-friendly channel of health information.

### Limitations and future research

Our study contributes to the understanding of digital clinical empathy, yet several limitations must be acknowledged. Firstly, our sampling strategy might have introduced self-selection bias, as participants responded to our recruitment efforts, primarily via cancer-related social media channels. Therefore, our sample might not be representative of the potential users (e.g., users with a suspected diagnosis of cancer or in the early stages of their cancer journey), limiting the generalizability of our findings. Moreover, although our sample included individuals with migrant backgrounds, discussions around empathy in the focus groups did not prominently feature issues specific to minority groups. However, language-related challenges observed during usability tests highlight the need for future research to delve deeper into the intersection of empathy and the experiences of minority groups, particularly in digital healthcare settings. Secondly, the usability test was staged and did not involve real emotionally charged queries. Participants were aware it was a test, which has influenced their reported experiences. Thirdly, the CIS provides multiple channels of communication, such as telephone and email. Our study focused only on the live chat feature, and the experience of empathy could differ when these features are used in combination. Future studies could benefit from exploring the synergistic effect of these channels and the unique characteristics of each medium, employing Grondin’s [18] conceptual framework. Fourthly, our study aimed to separate and analyze each of the three processes of empathy separately. Despite our efforts to individually analyze each, understanding, communicating, and acting upon users’ emotional states often interweave and should also be viewed holistically. Lastly, our study is qualitative in nature. While this provides rich, detailed data, it lacks the breadth of quantitative studies. A user survey is needed to confirm and quantify the patterns identified in this research. Moreover, examining the content of the chat interactions in future research could provide valuable insights into how specific language and word choices relate to the strategies employed to convey empathy in digital communication.

## Conclusions

Our study reveals that empathy remains a complex and nuanced concept, even more so in the digital realm. The absence of non-verbal cues and the reliance on written communication in live chats can hinder the accurate recognition, communication and enactment of empathy. Our study also underscores the spectrum of digital clinical empathy techniques and the diverse preferences among participants. Their emphasis on taking time to provide valuable information (i.e., laypeople-oriented while also fitting the digital communication channel) and being authentic in communicating with the user as a form of empathy is notable.

In conclusion, our study demonstrates that while the digital age has reshaped healthcare encounters, the fundamental need for empathy in health-related areas remains unchanged. The challenge lies in adapting traditional forms of empathetic communication to digital mediums. It is crucial for health professionals to be aware of the potential difficulties of each medium and to continuously strive to understand and address patients’ needs, especially in a text-based environment. As digital healthcare continues to evolve with AI technologies that are trained to imitate empathetic communication, so too must our understanding and practice of empathy within this realm.

### Electronic supplementary material

Below is the link to the electronic supplementary material.

Supplementary Material 1

## Data Availability

The datasets used during the current study are available from the corresponding author on reasonable request.

## Abbreviations

CIS: Cancer Information Service
FGP: Focus group participant
RQ: Research question
UTU: Usability test user
