# Supplementary material for: Digital clinical empathy in a live chat: multiple findings from a formative qualitative study and usability tests
Source: BMC Health Serv Res. 2024 Mar 8;24:314. doi: 10.1186/s12913-024-10785-8 (PMC10921626; doi:10.1186/s12913-024-10785-8)
Supplement: Supplementary file 1 — Supplementary Material 1 [file 12913_2024_10785_MOESM1_ESM.docx]

Question guide for interviews, focus groups and reflections with users and health professionals for a live chat with a topical focus on familial cancer

**Phase 1**

Focus groups with potential users

- Have you ever used a live chat with a company or so?
- If so, can you tell us about your personal experience? Why did you use live chat? How satisfied were you with the experience? Would you use the format again and if so, for what purpose?
- For what kind of questions and information do you think a live chat is appropriate? And for which ones is it not?
- The CIS already offers its services via email and telephone. What would be the function of the live chat offer compared to the already existing offers?
- What do you think are the limitations of a live chat for medical inquiries about cancer? What are its potentials?
- What is important to you in terms of the person answering questions in the live chat?

What makes the person credible? What would make them untrustworthy?

- What are your requirements for communication in the live chat so that you will feel you have made progress with your concern? Asking the other way around, how should communication not be done and why?
- Information can be communicated in different ways - from very factual to emotional, personal to humorous. Can you describe your preferences?
- Empathy is an important part of medical communication. How can these principles be applied in a chat format? What are potential challenges of empathetic communication?

Interviews with health professionals

- The CIS offers its services via email and telephone. A written live chat is different from a telephone call/group conversation. What does this mean for communication? (From the perspective of the enquirer and the respondent)
- How should communication in the live chat look like so that it helps the person making the inquiry in his/her concern?
- For what kind of questions and information do you think a live chat is appropriate? And for which ones is it not?
- Information can be communicated in different ways - from very factual, evidence-based to emotional, personal to humorous. Based on your experience, how would you communicate with the enquirer?
- What aspects are important when communicating with users? Asking the other way around, how should communication not be done?
- Empathy is an important part of medical communication and is anchored in the principles of CIS. How can these principles be applied in a chat format?
- What are potential challenges of empathetic communication in a live chat format?

**Phase 2**

Interviews after usability tests with users

- What is your general impression of the live chat?
- Please name three words that describe the application for you.
- How do you feel after the chat interaction? Is there anything you would have liked to know before you started the chat?
- Did you feel that your request was answered appropriately? What did you feel was missing in the response to your inquiry?
- How did you perceive the communication with the person answering at the other end?

How would you rate the responding person's communication style?

- Empathy is an important part of medical communication. To what extent was this achieved in the chat?
- What did you expect from your chat partner in terms of empathic communication? Were your expectations fulfilled?
- What are the advantages and disadvantages of written communication for the expression of empathy?
- Do you feel that the chat was the right format to adequately clarify your questions?
- Imagine that you have to make a decision regarding the topic you have just discussed in the chat. For example, for or against a certain treatment or intervention - do you know what you could do next to get more information or what can help you to make a decision?
- What did you like about using the live chat? What did you dislike about the chat and how could it be improved?

Questions for reflection with health professionals

- What are your thoughts about the two rounds of usability tests?
- To what extent did you feel that you were able to identify and respond to the concerns and needs of the users in the written contact?
- How did you feel about expressing empathy through the chat?
- What forms of empathy can be expressed in writing via chat?
- What are the advantages and disadvantages of written communication for the expression of empathy?
- What open concerns and difficulties do you see with regard to the upcoming launch of the chat?
